# Opioid-related deaths in Northern Ontario in the early COVID-19 pandemic period

**DOI:** 10.17269/s41997-024-00906-5

**Published:** 2024-07-29

**Authors:** Alexandra Nunn, Amanda M. Perri, Hilary Gordon, John P. D. Harding, C. K. Jennifer Loo, John Tuinema

**Affiliations:** 1 Algoma Public Health, Sault Ste. Marie, ON Canada; 2 Public Health Agency of Canada, Ottawa, ON Canada; 3 Middlesex-London Health Unit, London, ON Canada; 4 Fraser Health Authority, Surrey, BC Canada; 5 Provincial Health Services Authority, Vancouver, BC Canada; 6 Northern Ontario School of Medicine University, Sudbury, ON Canada; 7 Dalla Lana School of Public Health, University of Toronto, Toronto, ON Canada

**Keywords:** Opioid epidemic, Drug overdose, Drug users, Social determinants of health, Public health, Épidémie d’opioïdes, mauvais usage des médicaments prescrits, usagers de drogues, déterminants sociaux de la santé, santé publique

## Abstract

**Objectives:**

In the first year of pandemic measures, opioid-related deaths across Ontario’s (ON) 34 public health units (PHUs) increased by 60%. Death rates for all seven Northern ON PHUs were above the provincial average. This study describes and compares factors surrounding opioid-related deaths before and after pandemic measures were introduced, for Northern ON compared to the rest of ON.

**Methods:**

Aggregate data were provided for Northern ON and the rest of the province by the Office of the Chief Coroner/Ontario Forensic Pathology Services. Opioid-related deaths were cohorted by date of death for the year before and after pandemic measures were introduced on March 16, 2020. Chi-square tests were used to compare between cohorts and geographies to determine significant differences for each variable, and for dichotomized levels within variables. *P*-values < 0.05 were considered statistically significant a priori.

**Results:**

In Northern ON, the number of opioid-related deaths approximately doubled from the pre-pandemic cohort (*n* = 185) to the early pandemic cohort (*n* = 365). Compared to the rest of ON, higher proportions of deaths occurred in Northern ON among individuals who lived and died in private residences, among women (although the majority of decedents were male) and among individuals employed in mining, quarrying, and oil and gas industries. Compared to the pre-pandemic year, in Northern ON, higher proportions of opioid-related deaths involved fentanyl and stimulants as direct contributors, and the majority involved evidence of inhaled drugs.

**Conclusion:**

Differences between the circumstances of death in Northern ON and in the rest of ON suggest opportunities to tailor interventions.

## Résumé

**Objectifs:**

Au cours de la première année des mesures de lutte contre la pandémie, les décès liés aux opioïdes dans les 34 bureaux de santé publique (BSP) de l’Ontario ont augmenté de 60 %. Les taux de mortalité dans les sept unités de santé publique du Nord de l’Ontario étaient plus élevés de la moyenne provinciale. Cette étude décrit et compare les facteurs entourant les décès liés aux opioïdes avant et après l’introduction des mesures de lutte contre la pandémie, dans le Nord de l’Ontario par rapport au reste de la province.

**Méthodes:**

Les données pour le Nord de l’Ontario et le reste de la province ont été fournies par le Bureau du coroner en chef/Service de médecine légale de l’Ontario. Les décès liés aux opioïdes ont été classés par date de décès pour l’année précédant et suivant l’introduction des mesures de lutte contre la pandémie le 16 mars 2020. Des tests du khi carré ont été utilisés pour comparer les cohortes et les zones géographiques afin de déterminer les différences significatives pour chaque variable et pour les niveaux dichotomisés au sein des variables. Les valeurs *p* < 0,05 ont été considérées statistiquement significatives.

**Résultats:**

Dans le Nord de l’Ontario, le nombre de décès liés aux opioïdes a approximativement doublé entre la cohorte prépandémique (n=185) et la cohorte du début de la pandémie (n=365). Comparativement au reste de l’Ontario, des proportions plus élevées de décès sont survenus dans le Nord de l’Ontario chez les personnes qui vivaient et sont décédées dans des résidences privées, chez les femmes (bien que la majorité des personnes décédées étaient des hommes) et chez les personnes employées dans les mines, les carrières et les industries pétrolières et gazières. Comparativement à l’année prépandémique, dans le Nord de l’Ontario, des proportions plus élevées de décès liés aux opioïdes impliquaient le fentanyl et les stimulants comme contributeurs directs, et la majorité d’entre eux impliquaient des preuves de drogues inhalées.

**Conclusion:**

Les différences entre les circonstances des décès dans le Nord de l’Ontario et dans le reste de la province suggèrent des possibilités d’adapter les interventions.

## Introduction

Morbidity and mortality related to opioids have been a long-standing public health issue in Canada. Opioid-related harms associated with historically high rates of opioid prescribing in North America prompted regulatory changes that have shifted trends in overdose deaths in Canada in the last decade (Imtiaz et al., 2020). Since 2015, increases in opioid toxicity deaths in Ontario (ON) have not been associated with consumption levels of prescription opioids. The current crisis is fueled by a toxic, unregulated drug supply, including synthetic opioids and other drug types (Imtiaz et al., 2020). Addressing the opioid-related harms epidemic is complex because of the many contributing factors such as socio-economic determinants of health, stigma, and the high prevalence of mental health disorders among people with opioid use disorder (OUD) (Morin et al., 2017).

During the first 6 months of the COVID-19 pandemic in ON, the weekly number of opioid-related deaths increased 135% from the year prior (Gomes et al., 2021a). This may have been attributed to a combination of factors, including instability in the supply of substances, changes in the quality and potency of substances, increased price of substances, and effects of pandemic measures related to COVID-19 mitigation, such as isolation and reduced access to services that provide care to people who use drugs (PWUD) (Ali et al., 2021).

ON has 34 local Public Health Units (PHUs), seven of which comprise Northern ON: Algoma Public Health, North Bay Parry Sound District Health Unit, Northwestern Health Unit, Porcupine Health Unit, Public Health Sudbury and Districts, Timiskaming Health Unit, and Thunder Bay District Health Unit. Opioid-related death rates in Northern ON PHUs were disproportionately impacted by the pandemic compared to the rest of ON. From 2019 to 2021, the death rate per 100,000 increased 134% in Northern ON from 21.7 to 50.8, whereas the rate in the rest of ON increased 73% from 10.0 to 17.3 (Public Health Ontario, 2022).

Following a report that identified death rates above the provincial average for all Northern ON PHUs in the early pandemic period (Gomes et al., 2021b), the PHUs sought aggregated data for this analysis to better understand the disproportionate burden. The objectives were to describe and compare factors surrounding opioid-related deaths between pre-pandemic and pandemic cohorts in Northern ON and the rest of ON.

## Methods

Data were provided by the Office of the Chief Coroner/Ontario Forensic Pathology Services (OCC/OFPS) from a standardized investigative tool that coroners used during investigations of suspected opioid-related deaths to collect information from multiple sources including health records, family, bystanders, and emergency responders (Gomes et al., 2021b). An opioid-related death was defined as an acute intoxication/toxicity death resulting from the direct contribution of consumed substance(s), where one or more of the substances was an opioid, regardless of how the opioid was obtained (Gomes et al., 2021b). The likely mode of drug use was determined by the coroner based on death investigation findings and drug paraphernalia found at the scene (Gomes et al., 2021b).

Opioid-related deaths were aggregated for the seven Northern ON PHUs and the rest of ON. Opioid-related deaths that occurred from March 16, 2019, to March 15, 2020 were included in the pre-pandemic cohort. Deaths that occurred from March 16, 2020, to March 15, 2021 were included in the pandemic cohort.

In the descriptive analysis of demographics, risk factors, and circumstances surrounding the deaths, cell counts less than 5 were suppressed to prevent identifiability. Some cell counts greater than 5 were also suppressed to prevent identifiability through subtraction. Chi-square tests were used to compare between cohorts and geographies to determine statistically significant differences for each variable, and for dichotomized levels within variables. *P*-values < 0.05 were considered statistically significant a priori.

To maximize inclusion of available data, descriptive analyses and chi-squared tests were performed on all data points available per variable, excluding deaths in which the variable was missing at the time of analysis.

## Results

In Northern ON, the number of opioid-related deaths increased by 97% from the pre-pandemic cohort to the pandemic cohort (185 to 365), compared to the rest of ON which had a 70% increase in deaths (from 1352 to 2295).

### Characteristics of decedents

Between the two study years, there were no significant changes in the distribution of deaths by age group across the province. In Northern ON, like in the rest of ON, the 25- to 44-year age group continued to account for the largest proportion of deaths in the pandemic cohort (53.7%), followed by the 44- to 65-year age group (35.1%).

Across the province, the proportion of male decedents increased in the pandemic period. In Northern ON, males accounted for 70.4% of deaths in the pandemic period, compared to 63.2% the previous year. Although males accounted for the majority of cases, there was a significantly higher proportion of female decedents in Northern ON in the pandemic period, compared to the rest of ON (29.6% vs. 23.4%, Table 1).

**Table 1:** Characteristics of decedents and deaths, comparing the Northern Ontario (ON) pre-pandemic and pandemic cohorts, and the rest of ON pandemic cohort

Characteristics	Rest of ON pre-pandemic cohort (*n* = 1352)		Rest of ON pandemic cohort (*n* = 2295)		Comparison of rest of ON cohorts (*p*-value)^a^	Northern ON pre-pandemic cohort (*n* = 185)		Northern ON pandemic cohort (*n* = 365)		Comparison of Northern ON cohorts (*p*-value)^b^	Comparison of Northern ON and rest of ON pandemic cohorts (*p*-value)^c^
	***n***	**%**	***n***	**%**		***n***	**%**	***n***	**%**		
Age group					0.38					0.63	0.79
≤ 24	114	8.4%	162	7.1%	0.13	c	c	31	8.5%	0.89	0.33
25 to 44	718	53.1%	1234	53.8%	0.70	104	56.2%	196	53.7%	0.53	0.98
45 to 64	477	35.3%	836	36.4%	0.49	64	34.6%	128	35.1%	0.91	0.62
65 +	43	3.2%	63	2.8%	0.45	†	†	10	2.7%	0.21	1.00
Sex					< 0.01*					0.09	0.01*
Male	971	71.8%	1757	76.6%		117	63.2%	257	70.4%		
Female	381	28.2%	538	23.4%		68	36.8%	108	29.6%		
Geographical area			(*n* = 2315)^d^		0.18			(*n* = 359)^d^		0.34	< 0.01*
Rural area	108	8.0%	207	8.9%	0.32	†	†	17	4.7%	0.14	0.01*
Small urban centre	124	9.2%	171	7.4%	0.06	33	17.9%	65	18.1%	0.96	< 0.01*
Medium urban centre	131	9.7%	231	10.0%	0.78	49	26.6%	107	29.8%	0.44	< 0.01*
Large urban centres	966	71.4%	1679	72.5%	0.48	98	53.3%	170	47.4%	0.19	< 0.01*
Unknown	23	1.7%	27	1.2%	0.18	†	†	0	0%		0.04*
Living arrangements at time of death					< 0.01*					0.75	< 0.01*
Private dwelling	992	73.5%	1583	70.2%	0.03*	147	79.5%	278	76.4%	0.41	0.02*
Other collective dwelling	91	6.7%	204	9.0%	0.02*	10	5.4%	21	5.8%	0.86	0.04*
Homeless	173	12.8%	385	17.1%	< 0.01*	18	9.7%	34	9.3%	0.88	< 0.01*
Other	30	2.2%	28	1.2%	0.02*	†	†	†	†	0.99	0.25
Unknown	64	4.7%	56	2.5%	< 0.01*	†	†	†	†	0.18	< 0.01*
Unavailable	2		39					1			
Employment status at time of death					0.03*					0.09	0.04*
Employed	215	15.9%	294	13.0%	0.02*	31	16.8%	41	11.3%	0.07	0.35
Unemployed	674	49.9%	1215	53.9%	0.02*	77	41.6%	181	49.7%	0.07	0.14
Retired	28	2.1%	36	1.6%	0.29	†	†	12	3.3%	0.26	0.03*
Unknown	433	32.1%	711	31.5%	0.73	†	†	130	35.7%	0.33	0.11
Unavailable	2		39					1			
Industry of work at time of death	(*n* = 215)		(*n* = 294)		< 0.01*	(*n* = 31)		(*n* = 41)		0.01*	< 0.01*
Construction	65	30.2%	87	29.6%	0.45	†	†	14	34.1%	0.04*	0.55
Mining, quarrying, and oil and gas extraction	†	†	0	0.0%	0.12	5	16.1%	8	19.5%	0.71	N/A**
Retail trade	†	†	9	3.1%	0.88	†	†	6	14.6%	0.11	< 0.01*
Other services	81	37.7%	118	40%	0.09	13	41.9%	†	†	< 0.01*	< 0.01*
Other trades	21	9.8%	35	11.9%	0.21	5	16.1%	5	12.2%	0.63	0.96
Unknown	38	17.7%	11	3.7%	< 0.01*	†	†	†	†	0.74	0.02*
Unavailable			34								
Recent release from correctional facility (prior 4 weeks)					0.42					0.98	0.20
Yes	57	4.2%	83	3.7%	0.41	6	3.2%	13	3.6%	0.84	0.92
No	782	57.9%	1275	56.5%	0.41	95	51.4%	188	51.6%	0.95	0.08
Unknown	511	37.9%	898	39.8%	0.25	84	45.4%	163	44.8%	0.89	0.24
Unavailable	2		39					1			
Manner of death					< 0.01*					0.34	0.66
Accidental	1250	92.5%	2135	95.4%	< 0.01*	174	94.1%	333	96.2%	0.24	0.46
Suicide	65	4.8%	57	2.5%	< 0.01*	†	†	6	1.7%	0.12	0.36
Undetermined	36	2.7%	47	2.1%	0.28	†	†	7	2.0%	0.85	0.93
Unavailable	1		56					19			
Location of incident					< 0.01*					0.59	0.01*
Private residence	1015	75.2%	1591	70.5%	< 0.01*	147	79.5%	289	79.4%	0.99	< 0.01*
Outdoors	92	6.8%	169	7.5%	0.45	7	3.8%	28	7.7%	0.08	0.89
Hotel/motel/inn	59	4.4%	176	7.8%	< 0.01*	13	7.0%	21	5.8%	0.56	0.17
Rooming house	59	4.4%	148	6.6%	0.01*	9	4.9%	16	4.4%	0.80	0.11
Shelter/supportive living	26	1.9%	62	2.7%	0.12	†	†	†	†	0.77	0.03*
Public indoor space	37	2.7%	29	1.3%	< 0.01*	†	†	†	†	0.99	0.23
Other	55	4.1%	65	2.9%	0.05	5	2.7%	†	†	0.16	0.05
Unknown	7	0.5%	16	0.7%	0.49	†	†	†	†	0.63	0.34
Unavailable	2		39					1			
Intervention at the scene					0.06					0.01*	< 0.01*
No-one present to intervene	716	53.0%	1113	49.3%	0.03*	86	46.5%	159	43.7%	0.53	0.05
Individual present who could intervene	237	17.6%	400	17.7%	0.89	63	34.1%	91	25.0%	0.03*	< 0.01*
Resuscitation attempted	187	13.9%	310	13.7%	0.93	48	25.9%	78	21.4%	0.23	< 0.01*
Naloxone administered	113	8.4%	195	8.6%	0.78	29	15.7%	56	15.4%	0.93	< 0.01*
Unknown	397	29.4%	743	32.9%	0.03*	36	19.5%	114	31.3%	< 0.01*	0.54
Unavailable	2		39					1			
Likely mode of drug use based on evidence at the scene					< 0.01*					0.16	< 0.01*
Injection only	254	18.8%	311	13.8%	< 0.01*	22	11.9%	37	10.1%	0.53	0.06
Inhalation only	313	23.2%	756	33.5%	< 0.01*	47	25.4%	123	33.7%	0.05	0.95
Injection and inhalation	174	12.9%	313	13.9%	0.40	54	29.2%	83	22.7%	0.10	< 0.01*
Unknown	609	45.1%	875	38.8%	< 0.01*	62	33.5%	122	33.4%	0.98	0.05
Unavailable	2		40								

^*^The *p*-value is less than 0.05 and considered statistically significant

^**^The *p*-value could not be calculated, but data show a notable difference

^†^Cell counts less than 5 have been suppressed to prevent identifiability. Some adjacent cells greater than 5 have also been suppressed to prevent identifiability through subtraction

^a^This *p*-value pertains to the comparison of the rest of ON pre-pandemic cohort and the rest of ON pandemic cohort.

^b^This *p*-value pertains to the comparison of the Northern ON pre-pandemic cohort and the Northern ON pandemic cohort

^c^This *p*-value pertains to the comparison of the Northern ON pandemic cohort and the rest of ON pandemic cohort

^d^The denominator for this variable is different from the cohort size for other variables due to an inconsistency in data extraction. It is not possible to re-identify the cohort included in the other variables. However, this discrepancy is not expected to change the interpretation

In the rest of ON, people living in private dwellings accounted for most deaths in the pandemic period (70.2%), but significantly higher proportions of decedents lived in other collective dwellings (9.0%) or were homeless (17.1%) compared to the preceding year. These trends were not observed in Northern ON, where a significantly higher proportion of decedents lived in private dwellings (76.4%) in the pandemic period compared to the rest of ON (Table 1).

Half of decedents in Northern ON in the pandemic period were unemployed (49.7%) and there was no significant change over time. By contrast, in the rest of Ontario, significantly more decedents were unemployed in the pandemic period (53.9%) compared to the pre-pandemic period (49.9%). Among decedents who were employed at the time of death, people working in the construction industry accounted for the highest proportion of deaths (34.1% in Northern ON and 29.6% in the rest of ON). In the pandemic period, Northern ON had higher proportions of employed decedents in the mining, quarrying, and oil and gas extraction industry (19.5%) and in the retail trade (14.6%), compared to the rest of ON (0% and 3.1%, respectively).

### Circumstances of death

As in the rest of the province, most deaths in Northern ON were determined to be accidental in both the pre-pandemic (94.1%) and pandemic (96.2%) cohorts (Table 1).

More Northern ON residents died in private residences (79.4%) compared to the rest of ON (70.5%) during the pandemic period. In Northern ON, a greater proportion of deaths involved an individual present who could intervene, compared to the rest of ON (25.0% vs. 17.7%). However, between the pre-pandemic and pandemic periods, there was a significant decrease in the presence of another individual at the scene in Northern ON (34.1% to 25.0%) that was not observed in the rest of ON (17.6% to 17.7%). A significantly higher proportion of deaths in Northern ON involved resuscitation attempts (21.4%) and administration of naloxone (15.4%) (Table 1).

In Northern ON, over half of deaths involved evidence of inhalation (56.4% vs. 47.4% for the rest of ON). A higher proportion of deaths had evidence of both injection and inhalation (22.7%) compared to the rest of ON (13.9%). Over time, there was an increase in deaths involving inhalation only, to approximately one third of deaths across the province (Table 1).

### Substances contributing to opioid-related deaths

In all cohorts, most deaths involved fentanyl as a direct contributor (85.3% in Northern ON and 88.5% in the rest of Ontario in the pandemic period, Table 2). Both regions of the province had significantly higher proportions of deaths attributed to fentanyl in the pandemic period. Codeine was involved in a small proportion of deaths, but was more frequently identified in Northern ON (4.0%) compared to the rest of ON (1.4%).

**Table 2:** Substances contributing to opioid-related deaths, comparing the Northern Ontario (ON) pre-pandemic and pandemic cohorts, and the rest of ON pandemic cohort

	Rest of ON pre-pandemic cohort (*n* = 1351)		Rest of ON pandemic cohort (*n* = 2239)		Comparison of rest of ON cohorts (*p*-value)	Northern ON pre-pandemic cohort (*n* = 185)		Northern ON pandemic cohort (*n* = 346)		Comparison of Northern ON cohorts (*p*-value)	Comparison of Northern ON and rest of ON pandemic cohorts (*p*-value)
Opioids (direct contributor)											
Fentanyl^a^	1042	77.1%	1981	88.5%	< 0.01*	133	71.9%	295	85.3%	< 0.01*	0.09
Methadone	184	13.6%	224	10.0%	< 0.01*	32	17.3%	32	9.2%	0.01*	0.66
Morphine	103	7.6%	101	4.5%	< 0.01*	15	8.1%	22	6.4%	0.45	0.13
Hydromorphone	134	9.9%	118	5.3%	< 0.01*	16	8.6%	17	4.9%	0.09	0.78
Oxycodone	120	8.9%	101	4.5%	< 0.01*	13	7.0%	15	4.3%	0.19	0.88
Codeine	33	2.4%	32	1.4%	0.03*	6	3.2%	14	4.0%	0.64	< 0.01*
Other fentanyl analogue^b^	371	27.5%	60	2.7%	< 0.01*	27	14.6%	5	1.4%	< 0.01*	0.17
Heroin	56	4.1%	36	1.6%	< 0.01*	6	3.2%	†	†	0.04*	0.29
Buprenorphine	†	†	†	†	0.42	†	†	†	†	0.25	0.66
Other opioids^c^	28	2.1%	33	1.5%	0.18	†	†	†	†	0.43	0.37
Alcohol (direct contributor)	167	12.4%	296	13.2%	0.46	25	13.5%	47	13.6%	0.98	0.85
Stimulants (direct contributor)	683	50.6%	1288	57.5%	< 0.01*	88	47.6%	203	58.7%	0.01*	0.69
Methamphetamine	287	21.2%	564	25.2%	0.01*	59	31.9%	105	30.3%	0.71	0.04*
Cocaine	492	36.4%	939	41.9%	< 0.01*	56	30.3%	151	43.6%	< 0.01*	0.55
Other stimulants^d^	19	1.4%	45	2.0%	0.19	†	†	†	†	0.68	0.14
Benzodiazepines (direct contributor)	467	34.6%	1303	58.2%	< 0.01*	14	7.6%	31	9.0%	0.58	< 0.01*
Flualprazolam	30	2.2%	60	2.7%	0.39	0	0.0%	0	0.0%	N/A	N/A
Etizolam	77	5.7%	828	37.0%	< 0.01*	0	0.0%	11	3.2%	N/A**	< 0.01*
Flubromazolam	†	†	81	3.6%	< 0.01*	0	0.0%	0	0.0%	N/A	N/A
Benzodiazepines (detected)	88	6.5%	75	3.3%	< 0.01*	43	23.2%	185	53.5%	< 0.01*	< 0.01*
Flualprazolam	9	0.7%	7	0.3%	0.12	†	†	5	1.4%	0.73	< 0.01*
Etizolam	20	1.5%	98	4.4%	< 0.01*	†	†	60	17.3%	< 0.01*	< 0.01*
Flubromazolam	†	†	16	0.7%	0.02*	0	0.0%	9	2.6%	N/A**	< 0.01*

^*^The *p*-value is less than 0.05 and considered statistically significant

^**^The *p*-value could not be calculated, but data show a notable difference

^†^Cell counts less than 5 have been suppressed to prevent identifiability. Some adjacent cells greater than 5 have also been suppressed to prevent identifiability through subtraction

^a^Fentanyl estimates include fentanyl analogues

^b^Includes carfentanil

^c^Includes tramadol, oxymorphone, and hydrocodone

^d^Other stimulants include 3,4-methylenedioxymethamphetamine (MDMA), methylenedioxyamphetamine (MDA), amphetamine (in the absence of methamphetamine), methylphenidate, and pseudoephedrine

Across ON, there was a significant increase in the proportion of deaths attributed to a stimulant (58.7% in Northern ON and 57.5% in the rest of Ontario). Cocaine was the most common stimulant involved in deaths in Northern ON (43.6%) and the rest of ON (41.9%), and both regions experienced an increase in the proportion of deaths involving cocaine. In the pandemic period, methamphetamine continued to be more frequently identified in Northern ON deaths (30.3%) compared to the rest of ON (25.2%).

There was a significant increase in the proportion of deaths attributed to benzodiazepines as direct contributors in the rest of ON (34.6% to 58.2%), while in Northern ON there was a significant increase in benzodiazepines detected, but not contributing to death (23.3% to 53.5%). Benzodiazepines were less frequently identified as a direct contributor to death in Northern ON in the pandemic period compared to the rest of ON (Table 2).

## Discussion

Our findings demonstrate that the first year of the pandemic brought a disproportionate increase in deaths in Northern ON compared to the rest of ON. Results from this study highlight the importance of recognizing regional differences in the drug toxicity crisis, the opportunity to further engage industries in harm reduction, and the need to improve access to harm reduction and treatment services in Northern ON.

### Recognizing regional differences

The pandemic had a profound effect on social isolation, access to services, and risk-taking behaviour of PWUD (Galarneau et al., 2021). Prior to COVID-19, Northern PHUs already faced unique challenges in responding to the drug toxicity crisis, including geographical barriers to care (e.g., the need to travel long distances), limited access to evidence-based treatment (e.g., supervised consumption sites, SCSs; opioid agonist therapy, OAT), and limited health human resources (Eibl et al., 2015). In Northern ON, the crisis should be understood as one component of a syndemic of health and socioeconomic disparities that contribute to higher rates of premature mortality (Buajitti et al., 2019).

PHUs in Northern ON conduct routine surveillance of opioid-related harms using data from emergency medical services, emergency department visits, hospitalizations, and deaths, and provide harm reduction services. Some Northern ON PHUs have opioid alert systems, which provide timely information about circulating toxic drugs and related harms to service providers, people who are exposed to the unregulated drug supply, or the public. Our study indicated that there were significant changes in drugs and mode of consumption attributed to death from one year to the next, and between jurisdictions. This highlights the importance of maintaining local surveillance and issuing locally relevant alerts about changes in the drug supply and mode of use, ideally in consultation with target audiences, to have the greatest potential to mitigate acute harms from the unregulated toxic drug supply (Volpe et al., 2023). Recent survey data from British Columbia provide evidence to support this intervention, in that over two thirds of individuals who saw or heard a drug alert reported changing their substance use behaviour to be safer (Daowd et al., 2023).

In Northern ON, deaths in the pandemic period included a higher proportion of females compared to the rest of ON. Qualitative research from Ontario about women’s experiences of opioid use identified challenges including social alienation, violence, and isolation (Macleod et al., 2021). In another study, participants recommended having staff trained in trauma-informed practice, site protocols sensitive to gender dynamics, and women staff and staff with lived experience in positions of responsibility (Xavier et al., 2021).

A higher proportion of people living in Northern ON health regions identify as Indigenous (17.8%), compared to 2.0% in the rest of ON (Statistics Canada, 2023). Data on Indigenous identity of decedents were not available for this study; however, a 2023 report prepared by the Chiefs of Ontario (COO) and the Ontario Drug Policy Research Network (ODPRN) identified nearly a tripling of the annual rate of opioid-related deaths among First Nations people in Ontario from 2019 to 2021 (from 4.1 to 11.4 per 10,000). The rate of death in 2021 was more than seven times higher among First Nations people compared to non-First Nations people, and First Nations women were disproportionately impacted, with the rate of death 11.5 times higher than for non-First Nations women. Funding for First Nations‒led research, programs, and services is needed to address the root causes of the drug toxicity crisis in Northern ON communities (COO & ODPRN, 2023).

Our study identified two findings that suggest increased isolation among PWUD during the pandemic period in Northern ON. First, a significantly higher proportion of deaths occurred in private residences in Northern ON compared to in the rest of ON (79.4% vs. 70.5%). This may reflect the lack of access to supervised consumption services or the geographical isolation of PWUD in rural areas. Second, in Northern ON, there was a significant decrease in the presence of an individual who could intervene, compared to the year prior. This may reflect a disproportionate impact of pandemic measures on the isolation of PWUD in Northern communities. Both of these findings suggest a potential role for the promotion of (and research on the effectiveness of) phone or text-based services like the National Overdose Response Service (NORS), or the Brave app, which provide remote support for developing and initiating a rescue plan in the event of an overdose (NORS, 2023; Brave Technology Co-op, 2024).

### Enhancing industry engagement in health promotion

Half of decedents in Northern ON in the pandemic period were unemployed at the time of death. Among those who were employed, there was a significant increase from the year prior in the number and proportion of decedents employed in the construction industry (from <5 to 14 [34.1%]). Deaths among people in this employment group were overrepresented, given that in 2021, the construction sector accounted for 7.8% of employment in Northern ON health regions and 7.2% in the rest of ON (Statistics Canada, 2023).

Northern ON also had a significantly higher proportion of decedents employed in the mining, quarrying, and oil and gas extraction industry (19.5%) compared to the rest of ON (0.0%) in the pandemic period. The disproportionate impact on people in this industry is partially explained by the limited activity of this sector outside of Northern ON. In 2021, 5.6% of the labour force in Northern ON was in mining, quarrying, and oil and gas extraction industries, compared to 0.2% in the rest of ON (Statistics Canada, 2023).

In Ontario, people with a history of employment in the construction industry have been disproportionately impacted by opioid toxicity deaths (Gomes et al., 2022). Research from the United States suggests that opioid overdose rates are highest among occupations with greatest physical work demands and least access to paid sick leave (Shaw et al., 2020). Increasingly precarious work arrangements (e.g. on-call workers, contractors) adds pressure to work through pain, which may be mitigated by opioid use (prescribed or as self-medication) (Shaw et al., 2020).

In Ontario, the Occupational Health and Safety Act (OHSA) was amended in 2023 to require that naloxone be available in workplaces in which there is risk of an opioid overdose among workers. In 2022, the ODPRN published a report on *Lives Lost to Opioid Toxicity among Ontarians Who Worked in the Construction Industry*, calling for the construction industry to recognize the stigma around drug use, improve access to naloxone within workplaces and homes, raise awareness, and increase access to evidence-based treatment, pain management, and mental health supports (Gomes et al., 2022). Collaboration with community services and community drug strategies could strengthen the industry’s response. Upstream prevention of opioid use at workplaces can include recognizing, evaluating, and controlling workplace hazards that might contribute to acute or chronic injury, providing accommodation for injured employees or those undergoing OUD treatment (Shaw et al., 2020). Our analysis suggests that in Northern ON, these calls to action should also be addressed to the mining, quarrying, and oil and gas extraction industry, another sector that could be prioritized for support to implement the OHSA naloxone requirement.

### Improving access to harm reduction and treatment services

Our study found that most deaths across Ontario were accidental, with no notable difference between regions. In both regions, the proportion of deaths attributed to fentanyl was significantly higher in the first year of the pandemic compared to the previous year. This finding is consistent with studies that found increased toxicity and fentanyl concentrations through drug-checking services and urine screening in British Columbia and Ontario, due to a combination of factors including border closures, disruptions of supply chains, and changes in substance use behaviours (Morin et al., 2021; Tobias et al., 2023). Our findings also suggest that in both regions, inhalation was the predominant mode of drug use among decedents, and that the proportion of deaths involving injection decreased across the time periods.

We also found that during the pandemic period, fewer deaths across the province were associated with methadone, possibly related to decreased treatment access and diversion of prescribed methadone. Methadone toxicity is dependent on the tolerance of the user, and in Ontario, deaths from methadone have been linked to diversion, presence of other drugs, and underlying disease processes (Albion et al., 2010). In all cohorts, buprenorphine (prescribed as OAT under the name suboxone) was identified as a direct contributor in a very small proportion of deaths (data suppressed), suggesting that it continues to be a safer option for those who respond to it (Srivastava et al., 2017).

Although these trends are similar across the province, responsiveness to changes in the unregulated drug supply, trends in mode of use, and the accessibility of treatment options like OAT differ between urban and more rural or remote settings. In the first year of the pandemic, disruptions to treatment and reduced monitoring of individuals in OAT were associated with rural communities in Northern and Southwestern Ontario having the most notable increase in fentanyl positive urine screens among OAT patients (Morin et al., 2021). Compared to the rest of ON, residents of Northern ON have limited access to evidence-informed services for PWUD such as SCSs and safer supply programs.

The disproportionate increase in overdose deaths in Northern ON in the context of limited services underscores the importance of improving access to evidence-based interventions for the treatment of opioid use disorder and harm reduction, including OAT, take-home naloxone programs, supervised consumption sites, and drug checking services, and considering safer supply of medical-grade opioids as evidence emerges (Ivsins et al., 2020; Karamouzian et al., 2018; Kennedy et al., 2019; Mattick et al., 2009; McDonald & Strang, 2016).

Our findings suggest that planning for SCSs should consider the trend toward increased use of inhaled drugs, incorporate sex- and gender-based analysis to ensure that they meet the needs of women and gender-diverse people, and provide drug checking services that evolve in response to changing composition of the unregulated drug supply. Services should support Indigenous clients by incorporating guiding principles such as strengths-based approaches, community-determined programming, and cultural humility (Assembly of First Nations, 2019), as well as compassion and client self-determination (Henderson et al., 2023).

In addition, we found that a significantly higher proportion of people died in Northern ON despite receiving interventions such as resuscitation attempts and naloxone administration. Our data did not distinguish between health care provider and peer emergency responders, and further investigation is needed to understand this finding (e.g., to explore possible regional differences in willingness to call emergency services).

For Northern ON decedents in the pandemic cohort, resuscitation attempts and naloxone administration were frequent when an individual was present to intervene: out of the 91 such deaths, resuscitation was attempted for 78 (85.7%), and naloxone was administered for 56 (61.5%). This draws attention to the actions of first responders, the potential mental health impacts of witnessing and attending to drug toxicity deaths, and the potential need for enhanced services to support the informal practice of “spotting” (i.e., when PWUD witness other people using drugs and respond if an overdose occurs) (Perri et al., 2021).

### Limitations

Data up to March 2021 were only available in aggregate, so we could not perform case-level or multivariate analyses. Importantly, the dataset did not include variables that address socio-economic status, Indigenous identity, race, or ethnicity, which precluded analysis of how equity-deserving groups are impacted in Northern ON. We performed analyses on all variables, excluding cases whose data were unavailable at the time of analysis, assuming there was no systematic bias among decedents whose data were unknown or missing. Finally, more recent data suggest that 2021 was the pandemic year with the highest number of cases, and circumstances around deaths during the pandemic period may have continued to change beyond the end of the study period.

## Conclusion

In the first year of the COVID-19 pandemic, the number of opioid-related deaths accelerated in Ontario. Northern ON was disproportionately affected, with a mortality rate nearly three times higher than in the rest of ON in 2021. Differences between the circumstances of death in Northern ON and in the rest of the province suggest opportunities to tailor interventions to address the disproportionate burden of opioid-related mortality in Northern ON. Plans for increased access to treatment and harm reduction services in Northern Ontario should consider the following: the increasing use of inhaled drugs, incorporating sex- and gender-based analysis, providing drug checking services that respond to changing contaminants in the unregulated supply, and promoting and evaluating phone or text-based overdose response services and additional supports around “spotting.” Finally, industry-level calls to action should be extended to mining, quarrying, and oil and gas extraction industries.

## Contributions to knowledge

What does this study add to existing knowledge?

- Pre-pandemic, Northern Ontario was disproportionately impacted by opioid-related deaths. During the early pandemic period, the disparity was exacerbated by high relative increases in death rates.
- This study compares circumstances of opioid-related deaths in Northern Ontario to those in the rest of Ontario. Higher proportions of deaths occurred among individuals who lived and died in private residences, among women (although the majority were male) and among individuals in mining, quarrying, and oil and gas industries.
- Changing patterns of drug use were observed. The proportion of deaths attributed to fentanyl and stimulants increased, and most involved evidence of inhaled drugs at the scene.

What are the key implications for public health interventions, practice, or policy?

- This study confirms similarities and highlights differences in the characteristics of opioid toxicity deaths in Northern Ontario compared to in the rest of Ontario, identifying opportunities for unique intervention.
- Plans for increased access to treatment and harm reduction services in Northern Ontario should consider the following: the increasing use of inhaled drugs, incorporating sex- and gender-based analysis, providing drug checking services that respond to changing contaminants in the unregulated supply, and promoting and evaluating phone or text-based overdose response services and additional supports around “spotting.”
- Industry-level calls to action should be extended to mining, quarrying, and oil and gas extraction industries.

## Data Availability

Upon reasonable request.

## Code availability

Upon reasonable request.

## References

[CR1] Albion, C., Shkrum, M., & Cairns, J. (2010). Contributing factors to methadone-related deaths in Ontario. *The American Journal of Forensic Medicine and Pathology*, *31*(4), 313–319. 10.1097/PAF.0b013e3181ca4b1e 10.1097/PAF.0b013e3181ca4b1e 20081524

[CR2] Ali, F., Russell, C., Nafeh, F., Rehm, J., LeBlanc, S., & Elton-Marshall, T. (2021). Changes in substance supply and use characteristics among people who use drugs (PWUD) during the COVID-19 global pandemic: A national qualitative assessment in Canada. *The International Journal on Drug Policy, 93*, 103237. 10.1016/j.drugpo.2021.103237 10.1016/j.drugpo.2021.103237 PMC9759688 33893026

[CR3] Assembly of First Nations. (2019). First Nations health priorities to reducing problematic opioid use. Ottawa, Ontario. https://afn.bynder.com/m/1de9ba2a97305ca4/original/2019-AFN-Opioid-Strategy-Report.pdf. Accessed 28 Mar 2024

[CR4] Brave Technology Co-op. (2024). Brave overdose detection system: For communities tackling the overdose crisis. Vancouver, British Columbia. https://www.brave.coop/overdose-detection-system. Accessed 25 March 2024.

[CR5] Buajitti, E., Watson, T., Norwood, T., Kornas, K., Bornbaum, C., Henry, D., & Rosella, L. (2019). Regional variation of premature mortality in Ontario, Canada: A spatial analysis. *Population Health Metrics*, *17*(1), 9. 10.1186/s12963-019-0193-9 10.1186/s12963-019-0193-9 PMC6670187 31366354

[CR6] Chiefs of Ontario and Ontario Drug Policy Research Network. (2023). Opioid use, related harms, and access to treatment, among First Nations in Ontario annual update, 2013–2021. Toronto, ON: Chiefs of Ontario. https://odprn.ca/research/publications/opioid-first-nations-annual-update-2013-2021/. Accessed 28 March 2024.

[CR7] Daowd, K., Ferguson, M., Liu, L., Loyal, J., Lock, K., Graham, B., Lamb, J., McDougall, J., & Buxton, J. A. (2023). Awareness, predictors and outcomes of drug alerts among people who access harm reduction services in British Columbia, Canada: Findings from a 2021 cross-sectional survey. *BMJ Open*, *13*(5), e071379. 10.1136/bmjopen-2022-071379 10.1136/bmjopen-2022-071379 PMC10174008 37160395

[CR8] Eibl, J. K., Gomes, T., Martins, D., Camacho, X., Juurlink, D. N., Mamdani, M. M., Dhalla, I. A., & Marsh, D. C. (2015). Evaluating the Effectiveness of First-Time Methadone Maintenance Therapy Across Northern, Rural, and Urban Regions of Ontario, Canada. *Journal of Addiction Medicine*, *9*(6), 440–446. 10.1097/ADM.0000000000000156 10.1097/ADM.0000000000000156 PMC4629488 26484843

[CR9] Galarneau, L. R., Hilburt, J., O’Neill, Z. R., Buxton, J. A., Scheuermeyer, F. X., Dong, K., Kaczorowski, J., Orkin, A. M., Barbic, S. P., Bath, M., Moe, J., Miles, I., Tobin, D., Grier, S., Garrod, E., & Kestler, A. (2021). Experiences of people with opioid use disorder during the COVID-19 pandemic: A qualitative study. *PloS one*, *16*(7), e0255396. 10.1371/journal.pone.0255396 10.1371/journal.pone.0255396 PMC8320992 34324589

[CR10] Gomes, T., Iacono, A., Kolla, G., Nunez, E., Leece, P., Wang, T., . . . Watford, J. (2022). Lives lost to opioid toxicity among Ontarians who worked in construction. Ontario Drug Policy Research Network, Toronto, ON. https://odprn.ca/research/publications/opioids-in-the-construction-industry/. Accessed 18 Apr 2024

[CR11] Gomes, T., Kitchen, S. A., & Murray, R. (2021a). Measuring the burden of opioid-related mortality in Ontario, Canada, during the COVID-19 pandemic. *JAMA Network Open,**4*(5), e2112865. 10.1001/jamanetworkopen.2021.12865 34037734 10.1001/jamanetworkopen.2021.12865 PMC8155822

[CR12] Gomes, T., Murray, R., Kolla, G., Leece, P., Bansal, S., Besharah, J., . . . Watford, J. (2021b). Changing circumstances surrounding opioid-related deaths in Ontario during the COVID-19 pandemic. Ontario Drug Policy Research Network, Office of the Chief Coroner for Ontario and Agency for Health Protection and Promotion (Public Health Ontario), Toronto, ON. https://www.publichealthontario.ca/-/media/Documents/C/2021/changing-circumstances-surrounding-opioid-related-deaths.pdf?rev=3207bc61021c4b5d8cc48fe55ebb7e23&sc_lang=en. Accessed 18 Apr 2024

[CR13] Henderson, R., McInnes, A., Danyluk, A., Wadsworth, I., Healy, B., & Crowshoe, L. (2023). A realist review of best practices and contextual factors enhancing treatment of opioid dependence in Indigenous contexts. *Harm Reduction Journal*, *20*(1), 34. 10.1186/s12954-023-00740-x 10.1186/s12954-023-00740-x PMC10022548 36932417

[CR14] Imtiaz, S., Shield, K. D., Fischer, B., Elton-Marshall, T., Sornpaisarn, B., Probst, C., & Rehm, J. (2020). Recent changes in trends of opioid overdose deaths in North America. *Substance Abuse Treatment, Prevention, and Policy*, *15*(1), 66. 10.1186/s13011-020-00308-z 10.1186/s13011-020-00308-z PMC7457770 32867799

[CR15] Ivsins, A., Boyd, J., Beletsky, L., & McNeil, R. (2020). Tackling the overdose crisis: The role of safe supply. *International Journal of Drug Policy,**80*, 102769. 10.1016/j.drugpo.2020.102769 32446183 10.1016/j.drugpo.2020.102769 PMC7252037

[CR16] Karamouzian, M., Dohoo, C., Forsting, S., McNeil, R., Kerr, T., & Lysyshyn, M. (2018). Evaluation of a fentanyl drug checking service for clients of a supervised injection facility, Vancouver, Canada. *Harm Reduction Journal*, *15*(1), 46. 10.1186/s12954-018-0252-8 10.1186/s12954-018-0252-8 PMC6131768 30200991

[CR17] Kennedy, M. C., Hayashi, K., Milloy, M. J., Wood, E., & Kerr, T. (2019). Supervised injection facility use and all-cause mortality among people who inject drugs in Vancouver, Canada: A cohort study. *PLoS Medicine,**16*(11), e1002964. 10.1371/journal.pmed.1002964 31770391 10.1371/journal.pmed.1002964 PMC6879115

[CR18] Macleod, E. R., Tajbakhsh, I., Hamilton-Wright, S., et al. (2021). “They’re not doing enough.”: Women’s experiences with opioids and naloxone in Toronto. *Subst Abuse Treat Prev Policy*, *16*, 26. 10.1186/s13011-021-00360-3 10.1186/s13011-021-00360-3 PMC7980746 33743756

[CR19] Mattick, R. P., Breen, C., Kimber, J., & Davoli, M. (2009). Methadone maintenance therapy versus no opioid replacement therapy for opioid dependence. *The Cochrane Database of Systematic Reviews, 2009*(3), CD002209. 10.1002/14651858.CD002209.pub2 10.1002/14651858.CD002209 12519570

[CR20] McDonald, R., & Strang, J. (2016). Are take-home naloxone programmes effective? Systematic review utilizing application of the Bradford Hill criteria. *Addiction,**111*(7), 1177–1187. 10.1111/add.13326 27028542 10.1111/add.13326 PMC5071734

[CR21] Morin, K., Acharya, S., Eibl, J., & Marsh, D. (2021). Evidence of increased Fentanyl use during the COVID-19 pandemic among opioid agonist treatment patients in Ontario, Canada. *International Journal of Drug Policy*, *90*(103088). 10.1016/j.drugpo.2020.103088 10.1016/j.drugpo.2020.103088 PMC7834895 33385974

[CR22] Morin, K., Eibl, J., & Franklyn, A. (2017). The opioid crisis: Past, present and future policy climate in Ontario, Canada. *Subst Abuse Treat Prev Policy*, *12*(45). 10.1186/s13011-017-0130-5 10.1186/s13011-017-0130-5 PMC5667516 29096653

[CR23] National Overdose Response Service. (2023). All about NORS. https://www.nors.ca/about. Accessed 18 April 2024.

[CR24] Perri, M., Kaminski, N., Bonn, M., Kolla, G., Guta, A., Bayoumi, A. M., Challacombe, L., Gagnon, M., Touesnard, N., McDougall, P., & Strike, C. (2021). A qualitative study on overdose response in the era of COVID-19 and beyond: how to spot someone so they never have to use alone. *Harm Reduction Journal, 18*(1), 85. 10.1186/s12954-021-00530-3 10.1186/s12954-021-00530-3 PMC8339679 34353323

[CR25] Public Health Ontario. (2022). Interactive opioid tool. https://www.publichealthontario.ca/en/Data-and-Analysis/Substance-Use/Interactive-Opioid-Tool. Accessed 20 January 2023.

[CR26] Shaw, W. S., Roelofs, C., & Punnett, L. (2020). Work Environment Factors and Prevention of Opioid-Related Deaths. *American Journal of Public Health, 110*(8), 1235–1241. 10.2105/AJPH.2020.305716 10.2105/AJPH.2020.305716 PMC7349438 32552015

[CR27] Srivastava, A., Kahan, M., & Nader, M. (2017). Primary care management of opioid use disorders: Abstinence, methadone, or buprenorphine-naloxone? *Canadian Family Physician, 63*(3), 200–205. PMC5349718 28292795

[CR28] Statistics Canada. (2023). Census profile (table). 2021 census of population. Statistics Canada Catalogue no. 98–316-X2021001. Ottawa. Released November 15, 2023. https://www12.statcan.gc.ca/census-recensement/2021/dp-pd/prof/index.cfm?Lang=E Accessed 28 March 2024.

[CR29] Tobias, S., Grant, C. J., Laing, R., Lysyshyn, M., Buxton, J. A., Tupper, K. W., Wood, E., & Ti, L. (2023). What impact did the COVID-19 pandemic have on the variability of fentanyl concentrations in the Vancouver, Canada illicit drug supply? An interrupted time-series analysis. *BMJ Public Health,**2023*(1), e000197. 10.1136/bmjph-2023-000197 10.1136/bmjph-2023-000197 PMC11812719 40017876

[CR30] Volpe, I., Brien, R., Grigg, J., Tzanetis, S., Crawford, S., Lyons, T., Lee, N., McKinnon, G., Hughes, C., Eade, A., & Barratt, M. J. (2023). 'We don't live in a harm reduction world, we live in a prohibition world': tensions arising in the design of drug alerts. *Harm Reduction Journal, 20*(1), 3. 10.1186/s12954-022-00716-3 10.1186/s12954-022-00716-3 PMC9829230 36624508

[CR31] Xavier, J., Lowe, L., & Rodrigues, S. (2021). Access to and safety for women at supervised consumption services. Findings from a community-based research project. Canadian Mental Health Association. https://cmha.ca/wp-content/uploads/2021/04/Women-and-SCS-Report_FINAL-April-2021.pdf. Accessed 28 March 2024.

